# Feasibility of a Voice-Enabled Medical Diary App (SpeakHealth) for Caregivers of Children With Special Health Care Needs and Health Care Providers: Mixed Methods Study

**DOI:** 10.2196/25503

**Published:** 2021-05-11

**Authors:** Emre Sezgin, Garey Noritz, Simon Lin, Yungui Huang

**Affiliations:** 1 The Abigail Wexner Research Institute Nationwide Children's Hospital Columbus, OH United States; 2 Department of Pediatrics Nationwide Children's Hospital Columbus, OH United States

**Keywords:** children with special health care needs, care management, care coordination, voice-enabled mobile app, health information technology, voice assistant, voice interaction, mobile phone

## Abstract

**Background:**

Children with special health care needs (CSHCN) require more than the usual care management and coordination efforts from caregivers and health care providers (HCPs). Health information and communication technologies can potentially facilitate these efforts to increase the quality of care received by CSHCN.

**Objective:**

In this study, we aim to assess the feasibility of a voice-enabled medical diary app (SpeakHealth) by investigating its potential use among caregivers and HCPs.

**Methods:**

Following a mixed methods approach, caregivers of CSHCN were interviewed (n=10) and surveyed (n=86) about their care management and communication technology use. Only interviewed participants were introduced to the SpeakHealth app prototype, and they tested the app during the interview session. In addition, we interviewed complex care HCPs (n=15) to understand their perception of the value of a home medical diary such as the SpeakHealth app. Quantitative data were analyzed using descriptive statistics and correlational analyses. Theoretical thematic analysis was used to analyze qualitative data.

**Results:**

The survey results indicated a positive attitude toward voice-enabled technology and features; however, there was no strong correlation among the measured items. The caregivers identified communication, information sharing, tracking medication, and appointments as fairly and highly important features of the app. Qualitative analysis revealed the following two overarching themes: *enablers and barriers in care communication* and *enablers and barriers in communication technologies*. The subthemes included parent roles, care communication technologies, and challenges. HCPs found the SpeakHealth app to be a promising tool for timely information collection that could be available for sharing information with the health system. Overall, the findings demonstrated a variety of needs and challenges for caregivers of CSHCN and opportunities for voice-enabled, interactive medical diary apps in care management and coordination. Caregivers fundamentally look for better information sharing and communication with HCPs. Health care and communication technologies can potentially improve care communication and coordination in addressing the patient and caregiver needs.

**Conclusions:**

The perspectives of caregivers and providers suggested both benefits and challenges in using the SpeakHealth app for medical note-taking and tracking health events at home. Our findings could inform researchers and developers about the potential development and use of a voice-enabled medical diary app.

## Introduction

### Background

Children with special health care needs (CSHCN) are at an increased risk for chronic physical, developmental, behavioral, or emotional disorders and also require frequent health care services [1]. Examples of conditions associated with special needs include cystic fibrosis, cerebral palsy, Down syndrome, epilepsy, and uncontrolled asthma. Approximately 1 in 4 US households (23%) have at least one CSHCN [2]. Caring for CSHCN requires communication and coordination networks among caregivers and health care providers (HCPs). Such needs could be even higher for children with medical complexity, a subset of CSHCN with the most intensive medical needs who often require medical care provided in the home by caregivers and home nurses [3].

Regulatory and legislative work has been put forward to improve quality outcomes in the US health care system by redesigning the care system [4]. One approach is to reduce fragmentation in the health care system by adopting an integrated care model [5] or a patient- and family-centered care model, including care coordination, to facilitate health care services for all stakeholders, including families, patients, and providers [4,6]. This is in line with caregivers’ need to communicate and share information with providers and their need for assistance in navigating the care system and care coordination [7]. Health information technologies can play a key role in facilitating care coordination by providing the tools and integrated systems required to enable information sharing, communication, and remote patient assistance [4,8]*.*

With many care activities occurring at home, caregivers may need to keep track of the health events of their children and communicate this information with providers to receive timely and accurate care. Medical diaries have been useful to keep track of symptoms and medications and can be leveraged to keep HCPs informed of the patient’s condition, which may lead to reduced errors such as overprescribing [9]. In the domain of health information and communication technologies, current solutions are promising [10-13]. Fiks et al [14] reported that web-based care portals had a positive impact on parents of children with uncontrolled asthma for tracking health activities, medications, and medical goals through the portal. Shared care plans for children with medical complexities over a cloud-based platform were found to reduce information barriers and improve care [15]. Gentles et al [16] summarized that health care technology being used in pediatric care communications ensures continuity of care independent of location and institution (eg, from hospital to home) by supporting timely health tracking and reducing geographical limitations. Similarly, Baysari and Westbrook [17] found mobile apps to be useful in supporting care coordination and facilitating care communications. However, few care coordination mobile apps are available for complex care coordination, especially for CSHCN.

### Objective

To help in reducing fragmentation, improving timely capture of health events at home, and facilitating care coordination of complex care, we developed a voice-enabled mobile app prototype (SpeakHealth). Informed by a multistakeholder group [18], by the emerging literature on voice interaction in health care communication and management [19-21], and by technology use in care coordination [10,12], the SpeakHealth app was designed to assist caregivers in capturing health events in a timely and convenient manner and to enable sharing of these medical notes with other caregivers and providers to enhance communication. This study focuses on the technology needs and voice technology adoption of caregivers, and the feasibility of the SpeakHealth app (prototype), which was conceptualized in a previous study [18]. Our research question was “How feasible is a voice-enabled mobile app use as a medical diary for caregivers of CSHCN to enhance care management and coordination with providers?”

## Methods

### Overview

We employed a mixed methods design to develop a better understanding of the use of communication technology and voice-enabled apps by comparing and combining different perspectives collected through quantitative surveys and qualitative interviews in a single phase (convergent mixed methods) [22]. We conducted a web-based survey to understand the awareness and perception of caregivers of CSHCN toward communication technology use in child care. The survey was developed by coauthors and the stakeholder group. In parallel, we tested SpeakHealth through face-to-face interviews with caregivers who were recruited from among the survey participants. Finally, we interviewed HCPs (n=15) to better understand the value of information collected via the SpeakHealth app and its potential to be used in health care delivery.

### Recruitment and Study Setting

The study participants (caregivers and HCPs) were primarily recruited from the Complex Care Clinic at the Nationwide Children’s Hospital (NCH; Columbus, Ohio). NCH is one of the largest pediatric hospitals in the US Midwest. NCH uses the Epic electronic medical records system and MyChart patient portal, which are mentioned in this paper.

To increase our reach, caregivers were invited to participate in the interview and survey with a convenience sampling approach using email invitations through hospital networks (digital board announcement and email), word of mouth, hospital social media channels, and community partners. The inclusion criteria for participants were as follows: (1) being caregivers with children who had been diagnosed with a complex medical condition and (2) being smartphone users during the study period. Within the study period, we received responses from 86 caregivers through a web-based survey, and 10 caregivers participated in the face-to-face interviews. HCPs who serve CSHCN were invited to participate through invitations sent to their hospital email addresses. All interviews were held within the hospital campus. All participants were screened (questions about the child’s condition, age, and participant’s smartphone use were included) and consent was obtained on the web. The caregiver interview participants were compensated with a US $30 gift card. No compensation was provided to the web-based survey participants or the HCPs. This study received institutional review board approval from the NCH (institutional review board number: #00000231).

### Data Collection

After caregivers were screened and they provided consent, they completed a web-based survey about demographics and technology awareness (June-August 2019). Subsequently, they were invited to a face-to-face interview. A semistructured interview framework was followed (a sample set of guiding questions of the interviews is available in Multimedia Appendix 1). The interview protocol focused on daily life and communication technology use, app testing scenarios, and user perception assessment questions. The user perception assessment questions were informed by the technology acceptance model [23]. Overall, 10 caregivers participated in interviews that took approximately 1 hour. Furthermore, 2 researchers participated in the interviews: one moderated the interviews and the other operated as a note taker. All interviews with caregivers were audio recorded. In addition, observational notes were taken during the interviews by the moderating researchers.

Along with the interviews, we assessed the awareness of caregivers regarding communication technology through a broader perspective by conducting a web-based survey. Overall, 86 participants completed the survey, which included the face-to-face interview participants.

**Figure 1:**
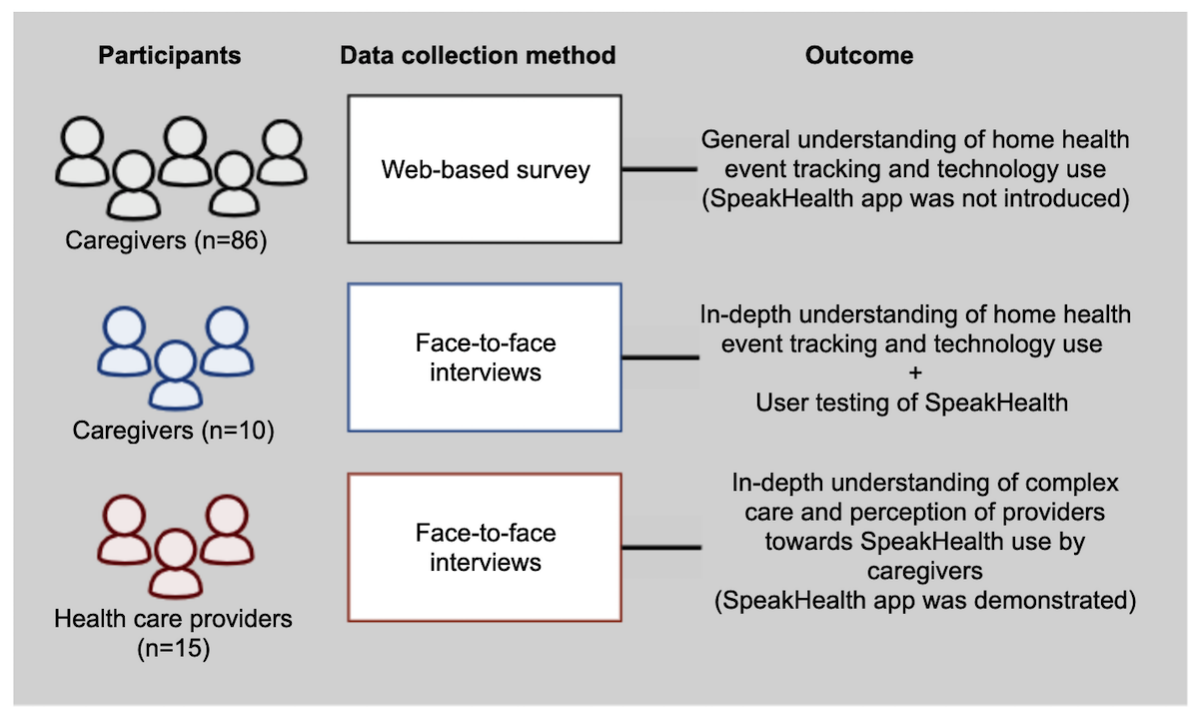
Study design and data collection.

HCPs were invited to a face-to-face interview where they were introduced to the project and the SpeakHealth app. The interviews were guided by two questions: (1) “What are your current experiences in complex care clinic?” (which focused on routine tasks, information needed, collected and missed information, and challenges) and (2) “How do you think the SpeakHealth app could help care management?” (which was used to understand if the information collected by the app fits the current care flow). Interviews with HCPs took approximately 40 minutes to complete. In total, 15 HCPs participated in the face-to-face interviews, which included physicians, nurses, care coordinators, and dietitians who have experience in the pediatric complex care clinic at the hospital where children or adolescents with chronic medical conditions (including neurodevelopmental disabilities and diseases affecting multiple organ systems) are being treated [24]. Figure 1 summarizes the study design and data collection process.

### App Prototype Development and Testing

**Figure 2:**
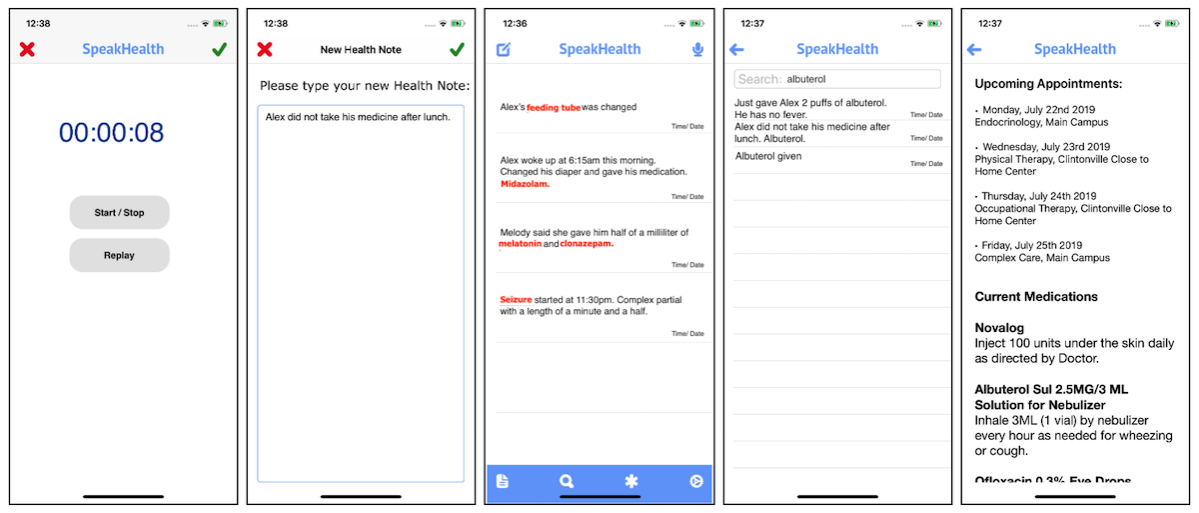
Screenshots of the SpeakHealth app. From left to right: voice interaction for note entry, note entry by text, notes overview and highlighted words, searching, and integration of medical information pulled from electronic health records.

The app prototype was designed based on the feedback of a multistakeholder group (including physicians, nurses, caregivers, care coordinators, digital health scientists and clinical informaticians, developers, and designers) at our hospital [18]. The prototype (Figure 2) was developed using Swift as an Apple (iOS) mobile app [25]. We used Amazon Web Services (AWS) application programmable interfaces (APIs) for the backend infrastructure and capabilities such as voice recognition and transcription (AWS Transcribe) [26]. The app leveraged existing voice technology components through iOS and AWS, and none of the voice interactive components or conversational agents were developed by the study team. The AWS infrastructure flowchart is presented in Multimedia Appendix 2.

Users were able to start the app through Siri (even when the phones were locked) by saying: “Hey Siri. Start SpeakHealth.” Then, SpeakHealth immediately started listening. After a speech segment was completed and collected, it was sent to AWS, transcribed, and sent back to the app and shared with the user. Users were also able to type notes or edit the transcription text.

As a part of the mock-up for planned features (not implemented with the prototype), users were able to see electronic health records (EHRs; eg, appointments and medications) and were able to access some of the prescribed medical care plans (eg, diabetes care guidelines) on the web. In addition, as a mock-up, important words and segments were highlighted in the notes (eg, medications and dosage). As they are not functional, we explained these features verbally to obtain participants’ feedback. However, we plan to implement EHR integration through patient portals and available APIs through EHR system providers to pull medications, appointments, and prescribed care plans. Note that highlighting will be implemented through natural language processing methods leveraging medical vocabulary and ontologies (eg, SNOMED [Systemized Nomenclature of Medicine] [27]).

Textbox 1 outlines the functional features (participants were able to test through the app) and introduced features (participants were informed about). Participants followed predefined scenarios to test the app (Textbox 2). We plan to include all the introduced features in the final production of the app.

**Tested and introduced features of SpeakHealth app.**

**Functional features**

- Voice-to-text note entry
- Text entry and editing
- Sham electronic health record (EHR) showing upcoming appointments and prescribed medications
- Preloaded information and guidelines
- Highlights of predefined notes

**Introduced (mock-up) features**

- Real-time note highlights
- Creating reports and sharing notes with health care providers
- Integration with EHRs
- Prescribed medical information and care plans

**App testing scenarios.**

**Scenario 0**

- This scenario is for the participants who did not use voice assistant before.
- Please say “Hey, Siri, how is the weather today?”, “How is traffic in downtown Columbus?”

**Scenario 1**

- Please say “Hey Siri, start SpeakHealth.” And, when you see the app running, say “just gave Alex 2 puffs of albuterol. He has no fever.”

**Scenario 2**

- Select one of the notes and edit the text. Then, please enter a new note with using “enter note” button, and type “Alex did not take his medicine after lunch.”

**Scenario 3**

- Check the last entered note. This is transcribed from your voice input. Please see the highlighted words. Those are identified as important to be shared with providers.

**Scenario 4**

- Use the search button to search for *albuterol* and check the results. You can see all your records where you administered albuterol.

**Scenario 5**

- See the user information content at the bottom left menu, find information about diabetes, and take a look at the content

**Scenario 6**

- See the medical record content at the bottom right menu and take a look at the content. Please find and check the next medical appointment date.

### Data Analysis

#### Qualitative Analysis

Audio recordings were transcribed verbatim and analyzed by the researcher, who moderated the interviews using Microsoft Office products. Observational notes taken during the interviews were used to supplement the transcripts, and these notes were incorporated into the transcriptions. Theoretical thematic analysis was used to systematically analyze the qualitative data [28]. Braun and Clarke [28] defined theoretical thematic analysis as a deductive method that is driven by the researcher’s theoretical or analytic interest in the research domain, and coding may focus on research questions.

In the initial step, the researchers read and reread the transcriptions to familiarize themselves with the data. During the reading iterations, instances were identified and labeled, and eventually coded. Codes were reviewed and gathered under potential themes, and an initial thematic map was created. Themes and codes were reviewed and the data were reviewed. The code groups and themes were revised, and the thematic map was updated. The codes and themes were reviewed by at least one researcher, and disagreements were resolved by a consensus among the researchers. Finally, the findings were reported and interpreted based on the literature findings. Coding and analysis of the qualitative data were completed using the Microsoft Office suite.

#### Quantitative Analysis

Survey responses were collected through the REDCap (Research Electronic Data Capture) web-based platform using Health Insurance Portability and Accountability Act–compliant secure services [29]. We completed a descriptive analysis of demographics and survey responses and conducted a correlational analysis. Data cleaning and verification were completed using Microsoft Excel, and the data were then analyzed using IBM SPSS software, version 26 (IBM Corporation).

## Results

### Survey Results

A total of 86 parents responded to the web-based survey. The responses are presented in Table 1. The survey participants were parents of at least one CSHCN with an average age of 11 (SD 7.4) years. Most children were diagnosed with developmental delay, speech and vision problems, epilepsy, intellectual disability, and learning disability. Most of them used daily prescribed medications (72/86, 86%); attended occupational, behavioral, or speech therapy (62/86, 74%); and used medical devices such as a feeding tube (45/86, 53%). Parents preferred to use a notebook (30/86, 35%) or paper to take notes when tracking symptoms, health events, and activities (29/86, 34%). The use of generic note-taking mobile apps was relatively low (13/86, 15%). Parents mainly tracked appointments (77/86, 90%) and symptoms (49/86, 57%) and set up reminders (54/86, 63%). Most parents reported that they always (25/86, 30%) or often (21/86, 38%) tracked all symptoms and health events.

**Table 1:** Survey responses.^a^

Variables		Values
**Child’s age (years; n=84)**		
	Range	0-31
	Mean (SD)	11.1 (7.4)
**Child’s diagnosed conditions (n=86), n (%)**		
	Developmental delay	56 (65)
	Speech problems	47 (55)
	Vision problems	44 (51)
	Seizures	38 (44)
	Intellectual disability	37 (43)
	Learning disability	36 (42)
	Cerebral palsy	33 (38)
	Joint or muscle problems	31 (36)
	Anxiety problems	23 (27)
	Hearing problems	23 (27)
	Asthma	22 (26)
	Behavioral problems	19 (22)
	Brain injury	17 (20)
	ADHD^b^	16 (19)
	Asperger, autism spectrum	11 (13)
	Depression	9 (10)
	Diabetes	3 (3)
	Other^c^	52 (60)
**What treatments or devices does your child use? (n=84), n (%)**		
	Daily prescribed medications	72 (86)
	Occupational, behavioral, or speech therapy	62 (74)
	Feeding tube	45 (53)
	Tracheostomy	18 (21)
	Other^d^	49 (58)
**How do you track symptoms, health events, and care activities at home currently? (n=85), n (%)**		
	Dedicated notebook	30 (35)
	Notes on paper or cards	29 (34)
	Health apps^e^	14 (16)
	Generic note-taking app	13 (15)
	I do not track	9 (10)
**What kind of care activities do you do for tracking? (n=86), n (%)**		
	Following appointments	77 (90)
	Setting up reminders	54 (63)
	Tracking symptoms	49 (57)
	Other^f^	17 (20)
**How often do you track symptoms and events at home in a day? (n=82), n (%)**		
	I always track and record all symptoms and events	25 (30)
	I often track and record symptoms and events	31 (38)
	I rarely track and record symptoms and events	16 (20)
	I do not track and record symptoms and events	10 (12)
**What do you think is the ideal tool or technology to use for tracking the symptoms and events at home? (n=85), n (%)**		
	Mobile phone and apps	65 (76)
	Pen and paper or notebook	38 (45)
	Tablet PC or iPad	31 (36)
	Laptop or PC	19 (22)
	Voice assistant (Amazon Alexa or Google Home)	13 (15)

^a^Responses to “other” are grouped by the most encounters that are not listed on the existing responses.

^b^ADHD: attention-deficit/hyperactivity disorder.

^c^Chronic lung disease, rare genetic diseases (DDX3X and complex vertebral malformation), neurodevelopmental diseases (hydrocephalus and bilateral schizencephaly), metabolic disorders, and heart disease.

^d^G tube, hearing aids, ventilation, ventriculoperitoneal shunt, wheelchair, nebulizer, suction machine, adaptive communication glucose and ketone monitor, and rescue inhaler.

^e^Calendar, Dexcom, seizure app, MyChart, Apple Health, and reminder app.

^f^Tube feed, medications, urine and bowel movements, blood glucose levels, seizures, medical changes, new words, and vitals.

Parents were also asked to identify the ideal communication technology or tool that they would prefer to use for tracking symptoms and events at home (Table 2). In contrast to the common method that they used (dedicated notebook and notes on a paper), most of them preferred to use mobile phones and apps for tracking (65/86, 76%) in addition to pen and paper (38/86, 45%). These were followed by tablet PCs (31/86, 36%) and laptops or PCs (19/86, 22%). The number of parents preferring voice technology as an ideal tool for tracking symptoms was relatively low (13/86, 15%). Finally, parents were asked to identify important features that a health app should have. Most of them found communication, sharing information, and tracking medication and appointments to be fairly important to very important (range 4.43-4.74). Health education and coaching for home care and voice interaction were found to be moderately important (range 3.28-3.72). Overall, parents found all the features to be important to very important and stated that such features would be a good fit to their current care routines and lifestyle (mean 4.6, SD 0.626).

**Table 2:** Ideal health app features.

How important is it for a health app to have the following features?	Participant, n (%)	Importance^a^, mean (SD)
Tracking medications	80 (93)	4.49 (0.994)
Tracking appointments	81 (94)	4.74 (0.787)
Hands-free and voice interactive engagement with the app	83 (97)	3.28 (1.355)
Keeping medical diary	83 (97)	4.25 (1.124)
Recording of audio or video to be shared with provider	83 (97)	3.96 (1.273)
Relevant health education or coaching contents for at-home care	83 (97)	3.72 (1.262)
Guidelines for coordinating care activities with other caregivers	82 (95)	4 (1.227)
Visualizing and summarizing the history of symptoms with statistics	81 (94)	4.22 (0.949)
Summary of home events between clinical visits to share with the provider	83 (97)	4.47 (0.738)
Interacting with personal health records at your provider (for instance, using the MyChart app)	82 (95)	4.57 (0.754)
Providing a communication channel to connect to the providers	82 (95)	4.43 (0.903)
Regarding your current lifestyle, habits and routines, what would be the impact of such a tool or technology if it had the features you ranked as *important* or above	82 (95)	4.6 (0.626)

^a^1: not at all important; 2: slightly important; 3: important; 4: fairly important; and 5: very important.

### Correlational Analysis

We used the Spearman nonparametric correlation test to explore if health condition, treatment, symptom tracking activity, tracking frequency, and expected app features were correlated. Our hypothesis was that having more health conditions and more treatments may positively correlate with tracking more health activities and an increase in the frequency of tracking. Similarly, they all may positively correlate with parents’ perceptions of more significant features in an ideal app. The variables tested for correlation were the child’s age, health condition (aggregated score), treatment (aggregated score), tracked activities (aggregated score), tracking frequency, and expected features (aggregated score). The missing data were excluded from pairwise deletion. However, we were unable to identify any strong correlations. There was a weak and positive correlation between the number of diagnosed conditions and total treatment (r=0.263; *P*=.02), tracking activities and total treatment (r=0.226; *P*=.04), and the child’s age and number of diagnosed conditions (r=0.247; *P*=.02).

### Interview Results

The interview participants were predominantly young (25-34 years: 4/10, 40%; 35-44 years: 4/10, 40%; and 45 or older: 2/10, 20%) and were predominantly part of a two-parent household (7/10, 70%) and White (10/10, 100%). Most households had 1 to 2 children (7/10, 70%), at least one of which had special needs (mean age 10, SD 8 years). The children had conditions with special health care needs for more than a year. Most children had multiple conditions. These conditions included the following: learning disability (4/10, 40%), speech problems (7/10, 70%), ADHD (1/10, 10%), depression (1/10, 10%), anxiety (3/10, 30%), diabetes (1/10, 10%), autism spectrum disorder (2/10, 20%), seizures (4/10, 40%), behavioral problems (2/10, 20%), hearing problems (1/10, 10%), developmental delay (6/10, 60%), vision problems (4/10, 40%), intellectual disability (6/10, 60%), joint or muscle problems (4/10, 40%), cerebral palsy (4/10, 40%), brain injury (3/10, 30%), genetic syndrome (2/10, 20%), and nonalcoholic fatty liver disease (1/10, 10%).

The children received daily prescribed medications (9/10, 90%), behavioral or speech therapy (6/10, 60%), feeding tube (5/10, 50%), tracheostomy (1/10, 10%), physical and occupational therapy (2/10, 20%), ventriculoperitoneal shunt (1/10, 10%), vagal nerve stimulator (1/10, 10%), and baclofen pump (1/10, 10%). Half of the parents often reported tracking health events at home, including activities such as tracking symptoms (8/10, 80%), follow-up appointments (10/10, 100%), and setting up reminders (6/10, 60%).

### Themes

#### Summary of Themes

We identified the following two overarching themes: *enablers and barriers in care communication* and *enablers and barriers in communication technologies*. These themes represent the perspectives of parent roles, needs, and communication dynamics with or without the technology component. The first theme, *enablers and barriers in care communication*, outlined the communication perspective in two subthemes. The first subtheme, *parent roles in care*, was an *enabler* theme, and it outlined the state of the parent-child relationship in terms of care delivery and management. This study aimed to understand the dynamics between parents and children. The second subtheme, *care communication challenges*, was a *barrier*, and it covered challenges encountered during child care. The second theme, *enablers and barriers in communication technologies*, outlined the communication perspective with the presence of technology in two subthemes. The first subtheme, *communication technology use in care*, was an *enabler*, and it explained the use of reported communication technologies (including voice-enabled technology) by caregivers. The second subtheme, *communication technology limitations*, was a *barrier*, and it was about the limitations of reported communication technologies (including voice-enabled technology).

#### Theme 1: Enablers and Barriers in Care Communication

##### Subtheme 1.1: Parent Roles in Care (Enablers)

Parents play a major role in the hands-on care of their children. They reported having scheduled tasks throughout the day, including feeding; administering medications; assisting in training and education; and scheduling medical care services, appointments, and activities. Most of the parents tracked medication supply, food intake, therapies, and medical appointments and set up reminders for recurring activities such as medication administration:

So, I usually get up in the morning and make sure her pills are out for her to take as she wakes up with some sort of food, because if she’ll take them on an empty stomach and then get sick....

Parent 1

...pretty much his biggest area is development, so just get on to therapies and things like that. Tracking just progress and you know, how much time in therapy we did each day is what we do.

Parent 2

For some parents, the frequency of each care activity and time spent may differ depending on the child’s condition. Some parents may need more guidance, assistance, and care coordination than others. For instance, some parents may require more time to prepare for feeding a child through a feeding tube. Other parents may need to help their children with after-school physical exercise. Similarly, a child’s age can potentially affect the effort dedicated by the parents. Children might be more independent regarding self-care as they age. However, their medical conditions may still affect their independence:

...we’re up at seven with my daughter. We hook her up to a feeding pump through a feeding tube and she gets several medicines in the morning...she gets four feeds throughout the day about every three hours. She also receives afternoon meds about three o’clock with her feed. At night, she gets several more medications with her evening feed.

Parent 3

##### Subtheme 1.2: Care Communication Challenges (Barriers)

Depending on the child’s condition and severity, families may encounter challenges that could lead to frustration and stress for the family. One of the overarching challenges is the *frequent need for medical communication*. Parents may need to communicate with multiple providers in a day and repeat the same information in each call:

He (child) probably has a dozen different care providers. So it’s never ending talking to all of them, keeping them up to date.

Parent 6

(Calling HCPs)...maybe a half dozen on average a week. However, there are some days, especially when he’s (child) sick, or we are transitioning or something. It (calling HCPs) could be six per day. I’d say six in a week to six in a day. Those busy days also involve like supplies and stuff too when we are calling to make sure food gets here and whatever.

Parent 6

In addition, parents reported the *difficulty of tracking* and the constant need to remember everything related to the child’s care. They may keep copies of health records and periodically keep notes on symptoms and medications. However, this may create some burden, and some information could still be missed. Parents come up with their own strategies to deal with this; however, the inefficiency of the tools used and methods for tracking and using information in communication were common challenges for parents:

...every time she eats she has to bolus for something or you know, every night she has to take a shot for a different type of shot to last throughout the day. So it’s just being able just to record quickly, we can record the sugars, but we can’t record how much we take.

Parent 5

Similar concerns were expressed by separated parents in tracking and sharing a child’s condition. Two of the parents, who were divorced, underlined the struggle of keeping all information at one place, as their child was not always staying at one home:

...me and her father are divorced. So if there was a way for us to both be able to log in and while she was gone, you know. If she complained of something, or if he had to take her to the ER, and I wasn’t there right away just to have who her doctors are, what her meds are, like basic major things that we could at least view as well as my husband. So kind of just like having that like information across the parents.

Parent 7

Parents understand the need and benefit of care coordination and communication; however, *gathering the information* to be communicated, such as the child’s health care notes and activities, was found to be difficult to keep up with:

...just tracking illness and stuff is just very difficult between different caregivers, different areas that he’s in. Care coordination is great in the fact that it gives appointments scheduled, but actually tracking symptoms of care is pretty difficult.

Parent 10

Children who need care assistance are not always compliant or cooperative with their caregivers. They may have trouble *gaining self-management* skills or may prefer not to keep track of health events and act based on personal preferences instead of meeting essential care needs, especially during the adolescent years:

They [HCPs] want to know so much information...telling a 14-year-old to write stuff down on paper. It’s not gonna happen.

Parent 5

#### Theme 2: Enablers and Barriers in Communication Technologies

##### Subtheme 2.1: Communication Technology Use in Care (Enablers)

Almost all parents had tablet computers or laptops with internet access at home; however, smartphones were the most frequently used personal technology. A couple of children used medical-grade technologies, such as glucose monitoring (Dexcom) and insulin devices (Omnipod), that parents are able to track through apps. However, some parents may prefer to use a pen and paper to keep track of health activities, such as medication refills, therapy appointments, and symptoms. This method is also regarded as convenient, with the use of color codes to follow multiple tasks. One of the methods to share care notes with other family members and home nurses is to use a calendar on a fridge:

Usually taking notes on calendars like...the one in the fridge. Sometimes I’ve heard her she feels weird or something write it on the fridge calendar.

Parent 4

Some parents mentioned the use of consumer-grade apps for tracking and note-taking, such as calendar apps and pharmacy apps. One parent kept a Microsoft Excel sheet to track and remember the data. As an mHealth app, some parents used MyChart (patient portal and mobile app of Epic EHR system) to check appointments, send messages, and check laboratory test results. Parents usually find apps through word of mouth, social media communities, and web-based searches. Similarly, information-seeking activities for child health were performed through web searches and community social media pages:

...have some of the Facebook groups that parent groups...what apps work for them? You know, and then that’s pretty much how I go about figuring things out. Sometimes I’ll go and look just like an app store search for some...[Parent 2]

there’s a lot of that diabetic Facebook groups that I’m in...I’m a kind of like a reader...[Parent 9]

With older children, parents expected their children to gain self-management skills. Parents envision children’s involvement in their medical care to be maintained with current communication technologies:

She (child) spends most of her time on her laptop, and her phone. I use my phone and laptop mostly. She also has an Apple Watch. And since she’s older, I think she could somehow be able to just “oh, I feel a weird” thing and tap on something and she also could record information.

Parent 1

##### Voice Interactions

Almost half of the parents reported that they were aware of and felt positive about using voice assistants via smartphones. Parents consider a mobile device to be an ideal tool for voice interaction and, when necessary, to track health events. Currently, some parents use voice interaction for basic tasks such as searching, sending reminders, using commands during driving, and using it for entertainment. Voice interaction was perceived as easier to use, especially for searching, setting up timers, or playing music. The use of voice in health communication received mixed feedback. Voice, as an enabler, was found to be a promising medium to receive medical information together as a family, instead of one parent taking the lead to get the information and share the child’s medical updates with the family. At home, medical communication through voice technology was not perceived as having a privacy risk:

...a lot of times what it is now is I’ll go look up something in my chart, look out for what the result is. I’ll go home tell whoever it is...tell her (child) about what they found or what they didn’t find and then tell what they said what they found...So it’s like cutting down like layers of happening to say the same information. Multiple times, if everyone’s sitting there they can all hear it together. And then that way, we are all on the same page at the same time together instead of staggered.

Parent 9

##### Subtheme 2.2: Communication Technology Limitations (Barriers)

Even though communication technologies have the potential to improve daily lives, finding the right one for an individual and their family, especially to meet the specific needs of health care could be burdensome. Parents mentioned problems in finding the right app, learning it, and using it regularly. Some parents stated that they did not have time to do so. In some cases, parents may have their own technological strategies:

I had to buy a little Bluetooth keyboard to go with the phone for when I do stuff on the phone, or the iPad. I was like, I gotta take this out. I can’t use my thumbs for everything.

Parent 8

Conversely, apps being used may not meet parents’ needs. One parent mentioned communicating digital notes was difficult because sending notes to the doctors from an app was troublesome. MyChart meets the basic needs, but parents may not *rely on it*. Some of the reasons were the issues with reminders and laboratory results, messaging and access limitations, and issues with reaching the messaging history and searching for notes. However, the reason may also be that parents may not know the functionalities of the app:

...I noticed sometimes when they’re (children) hospitalized, a lot of information doesn’t make it into MyChart anyhow. I don’t know why.

Parent 4

I feel like it’s like the one of the problems with MyChart is we have very little control. Yeah, I mean, would like to see there....

Parent 1

...here’s his certain idiosyncrasies of this thing or this process. If care providers can receive those notes in a centralized way. Okay, maybe MyChart already does that? I don’t know.

Parent 6

#### Limitations of Voice Interaction

Even though voice interaction was found to be promising, the need for a network connection to use voice interaction may be problematic. One parent mentioned their concern with poor internet connection in rural areas. Other concerns with voice interaction at home were proximity to the device (eg, being in the other room) and loud environments such as screaming children, playing instruments, or watching TV, which may disrupt voice interaction. In addition, individual technology interaction styles may differ and affect the extent of the use of voice, for example, one parent could prefer an auditory interaction, whereas another could prefer visual feedback.

Conversely, in terms of promoting a child’s self-management, the voice assistant’s capability in understanding commands was reported to be limited, especially in children with speech disorders:

...she has Down syndrome. Her speech is clear enough for you to understand but Siri or Alexa has a hard time understanding some things.

Parent 4

### App Feedback

Following the predefined scenarios (Textbox 2), participants tested the SpeakHealth app prototype and provided feedback. Parents mostly found voice-enabled interactive engagement to be useful for hands-free note-taking compared with typing. In particular, for parents who were not able to keep up with tracking health activities and taking notes, voice interaction was found to be promising to keep notes for use in care follow-ups:

...especially when sick, they want to know, okay, what is your sugar, when did she bolus and how much did she give? You know, what did she eat? And what time did they check it again? And how much [insulin] did you give again, and what’s your correction factor and all this other stuff. So, I mean, for us it (voice interaction) would be a total game changer because then we could just easily keep track because we are poor paper. We are poor paper charters. We just can’t do it. I don’t know why it’s just it’s so taxing to sit down and try to write down just all throughout the day, every day three o’clock in the morning, you’re not going to remember to write....

Parent 5

The main concerns about communicating personal health information through voice were as follows: (1) talking and listening out loud in public places and (2) voice recognition issues with understanding voice instructions and commands, especially with uncommon or medical words. An example of a misunderstanding of a command was experienced during the testing. While parents were narrating a medication name, *Albuterol* (an asthma medicine), it was understood as *computer-all* in two instances. However, interestingly, transcription errors were found to be acceptable as long as they were minimum:

...with using voice command, it’s often a matter of like, it seemed like the benefit outweighed the risk of it not hearing your particular word...I will use voice over typing it (note)

Parent 6

Parents also provided their feedback for the SpeakHealth app and voice interaction regarding how they would envision it to be used while caring for their children. Table 3 presents the breakdown of parents’ feedback regarding app and voice interaction.

**Table 3:** Parents’ feedback about the SpeakHealth app.

Feedback groups	App or voice interaction	Feedback summary
Sharing information	App	Parents like the idea of sharing notes with providers and significant others as well as easily notifying all health care providers in their circle in case of emergency. Shared accounts for everyone to access and edit notes received positive feedback.
Education	App	Educational links were found to be useful. Providing guidelines for first-time parents were suggested to be highly needed.
Integration	App	Electronic health record integration was well received. In addition, calendar integration was suggested. Parents would like to have more control over patient medical records in terms of access and transparency of all the notes.
Visualization	App	Parents would like to have a personalized dashboard, such as for epilepsy, seizure activities should be more visible. The highlighting ability was found to be useful.
Accessibility	Voice	Parents liked the idea of using voice while the phone was away and locked to enter notes. A parent suggested having the ability to navigate and search notes with voice.
Real-world use	Voice	Parents shared real-world scenarios to be used regarding their children’s condition. These include seizure timer, reminder of medications and appointments, searching medical notes, calling providers, and feeding tracking.
Voice-over typing	Voice	Parents stated that voice interaction helps to enter notes easier and faster than typing. Misunderstandings are correctable through text editing. Spell check feature for transcription was suggested for auto-correction.
More with voice: navigation	Voice	A parent mentioned navigation of notes with voice, search with voice.

### HCP Interviews

Most HCPs were pediatric physicians and nurses who were experienced complex care professionals. Table 4 summarizes HCP feedback.

Physicians in pediatric complex care frequently keep themselves up to date about their patient’s condition and maintain ongoing communication with each of the patients and their families. This requires them to periodically review a patient’s previous clinical notes, especially before visits. After visits, physicians may need 10-60 minutes to write patient visit notes. Physicians valued patient personal notes (eg, medical diaries and at-home care activities). They usually receive this information during visits or through phone triage notes. However, the integration of patient personal notes with the EHR requires a systematic effort to make information useful to physicians. In that regard, physicians would like to have this information *digestible* and *timely*, which means the presentation of the information should be succinct, organized, and accessible when it is needed. In addition, nurses emphasized communication efficiency. During periodic visits, emergency visits, or phone triage, the nurses need to receive information about the child’s condition and medication. This requires repeated effort from both sides (nurses and caregivers). Therefore, the solution is expected to make information exchange easier and more accurate, including information such as home care details, medication intake, and medication names. Similar feedback was provided by care coordinators, underlining the need for integrated medical information collection and sharing.

In the interviews, one example brought up by HCPs was seizure events. For tracking seizures, the patient- or caregiver-reported data are crucial in clinical decision-making. The frequency, duration, date, and trend over time are important information that must be accurately shared with HCPs. Therefore, keeping the information timely, categorizing by date, visualization with occurrence, duration, and time are some of the expectations from the patient data that are suggested for the SpeakHealth app. HCPs would also need to send a patient seizure action plan back to the caregiver to guide them in case of emergency.

Another example is gastrointestinal disorders in patients diagnosed with cystic fibrosis. Regarding the severity of the condition, significant time can be spent on the phone for guidance; information sharing; and collection of patient condition; feeding tube practice; and getting updates on symptoms, food intake, and bowel movements. A medical team, including nurses, physicians, dietitians, and pharmacists, have roles in clinical decision-making. Health events may occur multiple times a day, and the timely capture of patient personal notes can facilitate the decision-making process in terms of providing more accurate and complete data for assessment. For example, infant data information is collected through parent reports. Eventually, this information is entered into the EHR through necessary forms and templates (eg, nutrition plan and intake form) during triage or visits.

The SpeakHealth app was found to be promising for timely information collection that could be available for sharing with HCPs. It is suggested to have the ability to send reminders to parents for taking personal notes and eventually mapping these notes to the EHR.

**Table 4:** Health care provider feedback.

Profession	Expertise or specialty	Key takeaway
Physicians (n=7)	Internal medicine, neurodevelopmental disabilities, emergency medicine in pediatrics, gastroenterology, hepatology, nutrition, and pulmonary medicine	Organized, curated, categorized Visualization of the data Using patient notes into clinical notes Accessible over electronic health records
Nurses (n=4), care coordinators (n=2), clinical dietitian (n=1), and patient care pharmacist (n=1)	Complex care, pulmonary, and pediatric care	Medication adherence tracking and reminders Efficient communication and data sharing Reducing missing information

## Discussion

### Principal Findings

Our study demonstrated that caregivers of CSHCN may engage in different care activities and have different care management strategies depending on their child’s condition. Similar to the findings by Golden et al [7], caregivers desire better information sharing and communication with HCPs. Health care and communication technologies could potentially improve care communications and coordination [10]; however, a systematic approach is required to enable information to be conveniently captured at home and shared among caregivers and HCPs.

Our findings suggest that current communication technologies, especially mobile apps, could help to meet the fundamental care communication and coordination needs of at-home care, such as tracking medical symptoms, keeping up with medications and appointments, taking medical notes, accessing medical records, and communicating with HCPs. In line with this, voice interaction is perceived to be useful in taking medical notes. Even though voice technology adoption is still in the early stages of adoption by parents [30,31] and in health care [19], the use of voice in tandem with other methods (eg, audiovisual feedback through mobile phone enabling text entry) would increase voice technology adoption. As our findings suggest, taking medical notes is a significant part of care activity, and such efforts can facilitate care coordination activities by collecting necessary health information to be communicated with HCPs [7]. In that regard, having a medical diary conveniently at home is important as a part of the care strategy of parents. Considering the care activities that may keep a caregiver’s hands full, voice interaction can enhance and facilitate convenient note-taking and health event tracking. Currently, voice assistants (eg, Google Assistant, Amazon Alexa, and Apple Siri) running on smartphones and smart speakers (eg, Google Home and Amazon Echo) may still need improvement in the comprehension of medical terminology [20,32], health information exchange [19,32,33], and privacy and security measures [34] to provide health communication services.

Convenient note-taking and health event tracking are important; thus, having a shared platform among caregivers and HCPs is a significant step toward enabling patient note use (by multiple caregivers and providers) in shared clinical decision-making and improving quality of care. HCPs emphasized the value of timely health information collection, making it convenient for review through integration with medical records. Apps that are interoperable and designed to fit in the daily lifestyle of families and the clinical workflow of HCPs would help close the gap between communication and care coordination [13,17]. In the literature, integration of patient notes with the EHR has also been suggested to improve health outcomes [35]. Similarly, the OurNotes initiative (as a part of OpenNotes) values the ability of patients and caregivers to contribute to health records [36]. Therefore, the value and impact of medical notes kept at home would increase as they are shared with HCPs.

### Limitations and Future Studies

Our study has several limitations. We were not able to identify condition-specific needs or patient-provider experiences, but we were able to capture a broader perspective of perceptions. In addition, the study did not focus on the experiences of participants from different socioeconomic statuses or demographics. The participants of interviews and surveys were limited in number, and they were residents of Ohio, which may not be representative of the population as a whole. We tested a prototype during the interview sessions; therefore, we were not able to capture real-world evidence of app use. The accuracy of speech to text, performance of voice assistant, and their impact on interactions were not assessed systematically but planned for future phases. Future studies are planned to address assessing the usability and feasibility in real-world settings with a larger user group and investigating condition-specific needs. In addition, investigating the health system and implementation pathways would help to understand the feasibility of integration in the current health system. Such efforts will fill the gap in the health system and in the literature on medical note-taking and remote health tracking [10]. In addition, it may improve care coordination in terms of reducing the time spent on documentation, meeting care coordination needs, long-term care planning, and improving shared decision-making [2,13].

### Conclusions

In this paper, we present our findings on care management, coordination, and use of communication technology by caregivers at home, and we tested a voice-enabled medical diary app. Our study extends the literature in terms of understanding caregiver technology expectations and needs, as well as perceptions of HCPs. We believe the findings could further inform researchers and developers and technology policy makers in shared decision-making and patient-reported outcomes.

## Appendix

Multimedia Appendix 1 Interview guidelines.

Multimedia Appendix 2 SpeakHealth Amazon Web Services structure.

## Abbreviations

API: application programmable interface
AWS: Amazon Web Services
CSHCN: children with special health care needs
EHR: electronic health record
HCP: health care provider
NCH: Nationwide Children’s Hospital
REDCap: Research Electronic Data Capture
