# Supplementary material for: Feasibility of a Voice-Enabled Medical Diary App (SpeakHealth) for Caregivers of Children With Special Health Care Needs and Health Care Providers: Mixed Methods Study
Source: JMIR Form Res. 2021 May 11;5(5):e25503. doi: 10.2196/25503 (PMC8150418; doi:10.2196/25503)
Supplement: Multimedia Appendix 1 [file formative_v5i5e25503_app1.pdf]

Guiding questions for the interviews (sample set)

**Day in life**	• Can you summarize a regular day in your life - morning, work and school time and evening activities for you and your child?<br>• Routines for weekdays and weekends?
**Technology at home**	• What technologies do you use at home (TV, computer, smartphone)?<br>• Which apps do you use mostly at your phone?<br>• Which apps are your favorite ones?<br>• Have you used any apps related to care coordination or care management for your kid?<br>• If you could imagine a technology that would help better, what would you like to have?
**Voice technology**	• What do you think about using voice interaction with your Smartphone? Smart speaker?<br>• In what conditions do you use voice to interact with devices?
**Speakhealth app - utility focused questions**	• Do you think voice-interaction is an easy to use method compared the other apps you use? Why?<br>• Based on our previous scenarios, would you find it hard to enter your notes with voice in comparison to entering text?<br>• Do you think voice interaction could be useful to keep track of symptoms and medicine?<br>• Do you think voice-interaction is useful way to communicate health care with others?<br>• Would you use SpeakHealth or similar voice-interactive apps in the future to help you take care of your child? Why or why not?<br>• Do you have any recommendations for content or features that should be in the app for better care management and encouraging for you to use?<br>• Would you like the app/skill more if it was personalized or tailored more than its current state? If so, any suggestions?<br>• How would you envision the future use of voice interactive technologies for symptom tracking and care management?
