# Supplementary figures and images for: Feasibility of a Voice-Enabled Medical Diary App (SpeakHealth) for Caregivers of Children With Special Health Care Needs and Health Care Providers: Mixed Methods Study
Source: JMIR Form Res. 2021 May 11;5(5):e25503. doi: 10.2196/25503 (PMC8150418; doi:10.2196/25503)

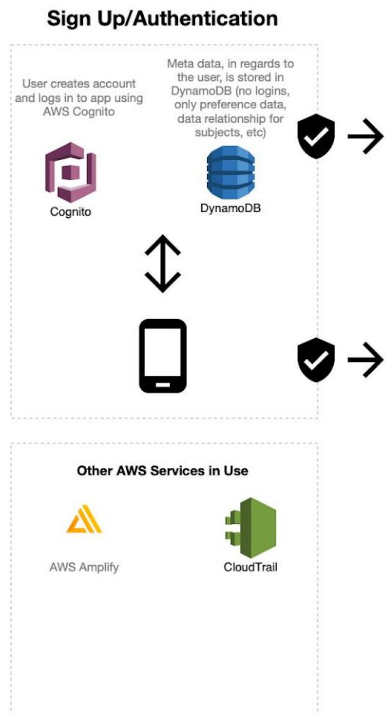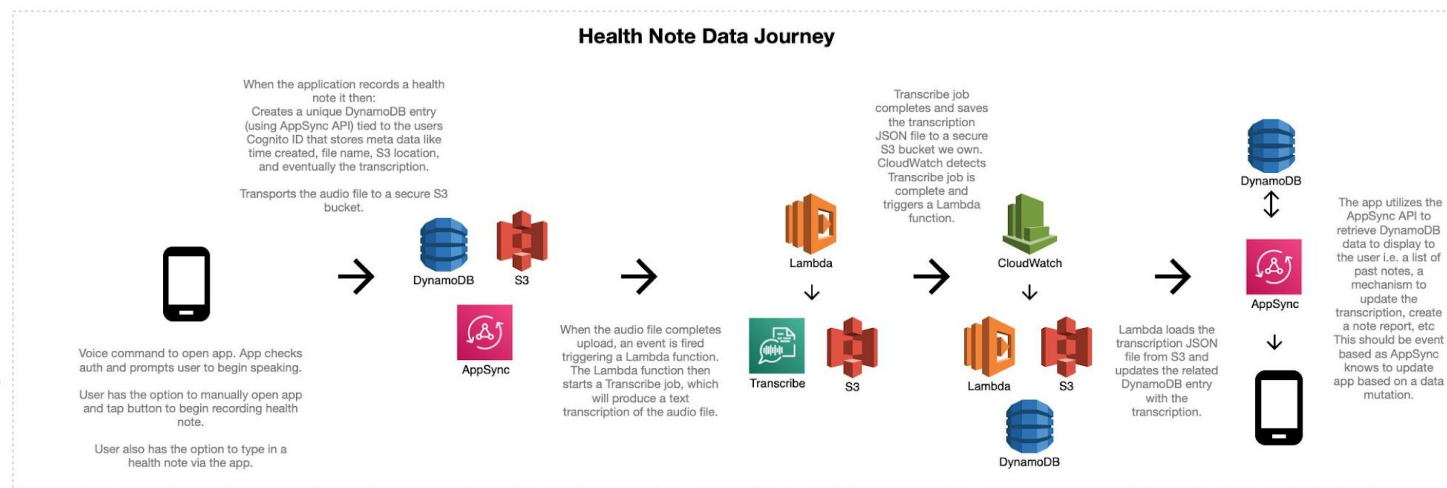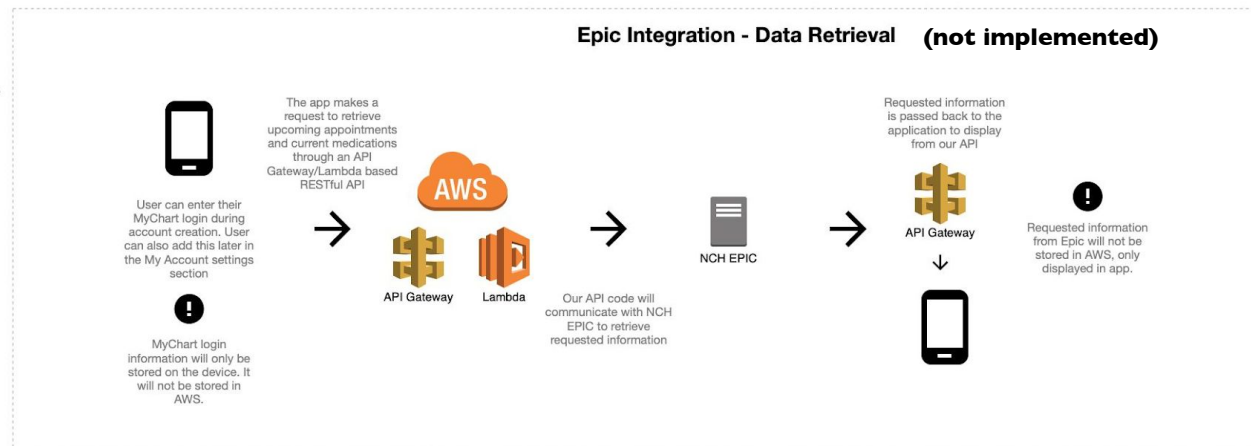

Supplement: Multimedia Appendix 2 [file formative_v5i5e25503_app2.pdf]
